# Laminin-332 promotes the invasion of oesophageal squamous cell carcinoma via PI3K activation

**DOI:** 10.1038/sj.bjc.6604252

**Published:** 2008-02-19

**Authors:** Y Baba, K-i Iyama, K Hirashima, Y Nagai, N Yoshida, N Hayashi, N Miyanari, H Baba

**Affiliations:** 1 Department of Gastroenterological Surgery, Graduate School of Medical Sciences, Kumamoto University 1-1-1 Honjo, Kumamoto City, Kumamoto 860-8556, Japan; 2 Department of Surgical Pathology, Kumamoto University Hospital, 1-1-1 Honjo, Kumamoto City, Kumamoto 860-8556, Japan

**Keywords:** laminin-332, PI3K pathway, oesophageal cancer, basement membrane, prognostic factor

## Abstract

Laminin-332 is major component of epithelial basement membrane, and has an important role in cell migration and tumour invasion. Recently, the phosphatidylinositol 3-kinase (PI3K) activation induced by laminin-332 during carcinogenesis or tumour invasion has been highlighted in skin squamous cell carcinoma. The expression of laminin-332 in 126 resected oesophageal squamous cell carcinoma (ESCC) specimens was immunohistochemically examined to determine its associations with the clinicopathological characteristics, and the effect of laminin-332 on the invasiveness and the PI3K activation was assessed by *in vitro* experiments using ESCC cell lines (ESCCs). Sections with immunostaining signals in >30% cancer cells, which were observed in 55 of 126 cases, were judged to be positive for laminin-332. The positivity was significantly correlated with pTNM stage and poor prognosis. Inactivation of the PI3K pathway by laminin-332 blocking antibody suppressed the invasiveness of TE8 cell line, which secreted laminin-332 at high level and had high PI3K activity. The addition of the purified laminin-332 activated the PI3K pathway and increased the invasiveness of TE11 cell line, which secreted laminin-332 at lower level and had low PI3K activity. The deactivation of PI3K pathway using the PI3K inhibitor decreased the invasiveness of ESCCs and the secretion of laminin-332 *in vitro*. The expression of laminin-332 was one of the prognostic factors of ESCC. Laminin-332 could provide the autocrine positive-feedback loop through PI3K activation, contributing the invasive ability. Therefore, the inhibitor of PI3K pathway might be useful as the anticancer therapies for ESCC.

Laminins are the best-known member of a family of the basement membrane (BM) protein, and they are important in cell growth, differentiation, adhesion and migration. The laminin molecule is a cross-shaped heterotrimer consisting of one heavy *α* chain and two light chains, *β* and *γ* chains (Timpl, 1989; Beck *et al*, 1990; Yurchenco and Schittny, 1990; Engle, 1992). To date, 16 laminin isoforms with different contributions of *α*1–*α*5, *β*1–*β*3, *γ*1–*γ*3 chains have been identified (Miyazaki, 2006; Marinkovich, 2007). Each laminin chain contains many functional domains, allowing the laminins to interact with various molecules in the extracellular matrix. For example, laminin *α* chains (*α*1–*α*5) contain large globular G domains in their C terminus, which is divided into five homologous subdomains (G1–G5). The G domain is thought to be a major site of interaction with specific receptors on the cell surface, including integrins, syndecans and *α*-dystroglycan. The N-terminal region of the three chains contains functional domains that are mainly involved in matrix assembly (Timpl, 1989; Beck *et al*, 1990; Yurchenco and Schittny, 1990; Engle, 1992).

Laminin-332 (previously known as laminin-5), which comprises *α*3, *β*3 and *γ*2 chains, is a major component of epithelial BM, acts as an adhesion substrate for the epithelial cells and regulates epithelial cell migration during epithelial regeneration and repair processes in normal tissues (Carter *et al*, 1991; Rousselle *et al*, 1991; Ryan *et al*, 1996). Laminin-332 interacts with two major epithelial integrin *α*3*β*1 and *α*6*β*4, and promotes the formation of two separate types of attachment structure, focal adhesion and stable anchoring contacts (Carter *et al*, 1990). In several types of carcinomas, laminin-332 has been reported to be highly expressed and to correlate well with poor patient prognosis (Pyke *et al*, 1995; Soini *et al*, 1996; Koshikawa *et al*, 1999; Matta *et al*, 1999; Ono *et al*, 1999; Yamamoto *et al*, 2001; Fukai *et al*, 2005). Laminin-332 is known to support cellular survival, proliferation and migration by activating many signal mediators through the ligation with *α*3*β*1 and *α*6*β*4 integrin, and to have important roles in tumour invasion and metastasis (Nguyen *et al*, 2000; Kariya and Miyazaki, 2004; Nikolopoulos *et al*, 2005). Particularly, the phosphatidylinositol 3-kinase (PI3K) activation induced by laminin-332 during carcinogenesis or tumour invasion has been highlighted (Marinkovich, 2007; Waterman *et al*, 2007). Marinkovich has demonstrated that the interaction of laminin-332 with *α*6*β*4 integrin and epidermal growth factor receptor (EGFR) was involved in promoting the PI3K activation and tumour invasion (Marinkovich, 2007).

Oesophageal squamous cell carcinoma (ESCC) is one of the most aggressive malignant tumours, and the prognosis of ESCC is worse than that of other digestive tract carcinomas (Enzinger and Mayer, 2003). As to ESCC, there are two papers demonstrating that the immunohistochemically increased cytoplasmic expression of laminin-332 is correlated with poor prognosis (Yamamoto *et al*, 2001; Fukai *et al*, 2005). However, to our knowledge, there are no data showing the relationship between laminin-332 and cancer cell invasiveness, using oesophageal squamous cancer cell lines (ESCCs).

The aims of this study are (1) to investigate the immunohistochemical expression of laminin-332 in ESCC and to evaluate the relationships between the clinicopathological characteristics and survival, and (2) to examine the biological role and the signalling pathway of laminin-332 using ESCCs.

## MATERIALS AND METHODS

### Patients

The present study involved 126 consecutive patients with ESCC who underwent the curative surgical resection at Kumamoto University Hospital from January 1997 to December 2006. All of these patients underwent an oesophagectomy with lymph node dissection. None of these patients underwent endoscopic mucosal resection, palliative resection, preoperative chemotherapy or radiotherapy, and none of them had synchronous or metachronous multiple cancers in other organs. Clinical data, including age, sex, tumour location, lymph node metastasis, T classification (Sobin and Wittekind, 2002), TNM stage (Sobin and Wittekind, 2002) and histological grading (Hamilton and Aaltonen, 2000), were available for all 126 patients. Patients were periodically (every 1–3 months) examined on an outpatient basis to make sure they did not have disease recurrence. The mean follow-up period for the 126 patients was 29.6 months (range, 1–110 months). Informed consent for the research was obtained from each patient. The study design was approved by the ethic review board of our university.

### Immunohistochemistry

Sections of 5 *μ*m in thickness were dewaxed in xylene and rehydrated in ethanol and then heated to 132°C in an autoclave for 5 min in 10 nmol l^−1^ citrate buffer (PH 6.3). The endogenous peroxidase activity was suppressed by a solution of 3% hydrogen peroxide in methanol for 60 min. The sections were immersed in 5% normal horse serum in PBS for 30 min, covered with mouse anti-laminin *γ*2 chain (1 : 200, Chemicon, Billerica, USA), and incubated overnight at 4°C. Negative controls were stained with PBS and normal mouse IgG instead of the primary antibody. Immunoreactions were performed using a Vecstatin peroxidase ABC kit (Vector Laboratories, Peterborough, UK). The antigenic sites were demonstrated by reacting the sections with a mixture of 0.05% 3,3-diaminobenzidine tetrahydrochloride (Dojindo Chemicals, Dojindo Laboratories, Kumamoto, Japan) in 0.05% mol l^−1^ Tris-HCl buffer, pH 7.6, containing 0.01% H2O2 for 5 min. The nuclei were stained with haematoxyline.

Sections with immunohistochemical staining in >30% of carcinoma cells at the invasive front were judged to be positive for laminin-332 as with the previous reports (Yamamoto *et al*, 2001; Fukai *et al*, 2005). The staining assessment was independently carried out by two of the authors (KI and YB) without any knowledge of either the clinical or survival data.

### Cell culture

ESCCs were obtained from Cell Resource Center for Biomedical Research, Tohoku University, Japan. Cell cultures were grown in recommended medium with 10% fetal bovine serum (FBS) and incubated in 5% CO_2_ at 37°C.

### Antibodies and other reagents

Antibodies for western blot analysis were as follows: rabbit anti-phospho Akt, rabbit anti-Akt (Cell Signaling Technology, Danvers, USA), mouse anti-laminin *γ*2 chain (Chemicon), mouse anti-epidermal growth factor receptor (ZYMED Laboratories, San Francisco, USA), mouse anti-integrin *β*4 (Santa Cruz Biotechnology, Santa Cruz, USA). Anti-laminin *γ*2 antibody (Chemicon) was used as the blocking antibody as with previous article (Decline and Rousselle, 2001). The purified human laminin-332 was purchased from Biodesign Internatinal, and the PI3K inhibitor Wortmannin was purchased from Santa Cruz Biotechnology.

### Western blot analysis

Cultured cells were solubilised with the lysis buffer (Tris-HCl (pH7.4). 25 mM, NaCl 100 mM, EDTA 2 mM, TritonX 1% with 10 *μ*g ml^−1^ Aprotinin, 10 *μ*g ml^−1^ Leupeptin, 1 mM Na_3_VO_4,_ 1 mM PMSF). To evaluate the secreted laminin-332 in the cell culture medium, each cell line was plated at 1 × 10^6^ cells per 100-mm culture dish in serum-free medium. Conditioned medium was collected at 24 h. 10 *μ*g of each protein sample was mixed with 4 × sample buffer including 10% *β*-mercaptoethanol and boiled 5 min. Next, equal amount of samples were separated on 7.5% (for the reduced laminin-332), 6.0% (for the non-reduced laminin-332) or 12% (for others) SDS–polyacrylamide gels and transferred to PVDF membranes. The membranes were probed with each primary antibody overnight at room temperature, followed by a 1 : 1000 dilution of peroxidase-conjugated IgG antibody (Sigma, St Louis, USA). Detection was accomplished with an ECL reagent (Amersham, Buckinghamshire, UK).

To evaluate the relationship between the secretion of laminin-332 and the PI3K pathway, the confluent cells were incubated with the PI3K inhibitor (Wortmannin) at different concentration for 24 h, and the culture medium was collected. Equal amount of samples were separated on 7.5% SDS–polyacrylamide gel and transferred to PVDF membranes.

### Cell-invasion assay

*In vitro* cell invasion assay was conducted using Biocoat Matrgel Invasion Chambers (BD Biosciences Discovery Labware, Franklin, USA) according to the manufacturer's protocols. After detaching the cells in 0.25% trypsin and counting, cells were diluted to 100 000 cells per ml in medium counting 0.5% FBS. A total of 500 *μ*l of cells were then placed in quintuple in the top chamber of the insert above medium counting 10% FBS, and were incubated in 37°C, 5% CO_2_ for 22 h. Next, the cells on the upper surface of the membrane were removed with a cotton swab, and the migrated cells on the underside of the membrane were fixed and stained using toluidine blue and counted in five random fields at × 200 magnification.

To examine the effects of the purified laminin-332, anti-laminin-332 blocking antibody and the PI3K inhibitor (Wortmannin) on the invasiveness of ESCCs, the purified laminin-332 (0.5 *μ*g ml^−1^), anti-laminin-332 blocking antibody (40 *μ*g ml^−1^) or the PI3K inhibitor (Wortmannin; 100 nM) was added into the top chamber. As controls, the purified albumin (0.5 *μ*g ml^−1^) or anti-IgG antibody (40 *μ*g ml^−1^) was used.

### Statistical analysis

Laminin-332 expression in ESCC was assessed to identify any associations with clinicopathological parameters using *χ*^2^ two-tailed test or Fisher's exact test. The disease-free survival and overall cancer-specific survival curves were constructed using the Kaplan–Meier method, and the log-rank test was used to evaluate the statistical significance of the differences. The prognostic significance of clinicopathological parameters was determined using univariate and multivariate Cox regression analysis. *In vitro* assay, a statistical analysis was performed using Mann–Whitney′s *U*-test for unpaired samples. Statistical analysis was performed using SPSS software (version 13 for Windows; SPSS, Chicago, IL, USA). A two-sided significance level of *P*<0.05 was used for all statistical analyses.

## RESULTS

### Immunohistochemical expression of laminin-332 in oesophageal squamous cell carcinomas

**Figure 1:**
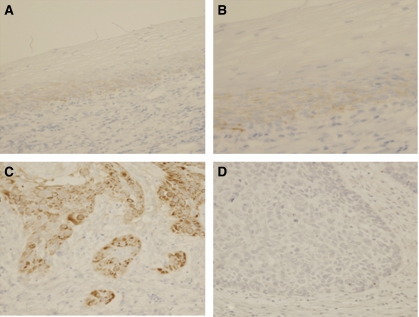
Immunohistochemical staining of laminin-332. Original magnification: × 200 (**A**,**C**,**D**) × 400 (**B**). (**A**, **B**) Expression of laminin-332 in normal oesophageal epithelium. Laminin-332 is expressed faintly in the cytoplasm of epithelial cells in the basal layer. (**C**) Positive staining of laminin-332 of ESCC is indicated. Laminin-332 is clearly shown in the cytoplasm of ESCC. (**D**) Negative staining of laminin-332 of ESCC is indicated.

In normal oesophageal epithelium, laminin-332 *γ*2 chain was stained weakly in the cytoplasm of epithelial cells near basal layer. (Figure 1A and B) In some cancer tissues, the cytoplasm of cancer cells was stained for laminin-332 *γ*2 chain at levels much stronger than those in normal BM. Sections with immunostaining signals in >30% of carcinoma cells, which were observed in 55 (44%) cases, were judged to be positive for laminin-332 *γ*2 chain expression (Figure 1C and D).

The relationships between laminin-332 expression and clinicopathological characteristics are summarised in Table 1. The expression of laminin-332 was significantly correlated to TNM stage (*P*=0.036). The patients with laminin-332-positive ESCC had a higher tendency to have lymph node metastasis and deep invasion than those with negative; however, the differences were not statistically significant (*P*=0.14 and *P*=0.15, respectively). On the other hand, there were no significant relationships between laminin-332 expression and age, sex, tumour location and histological grading.

**Figure 2:**
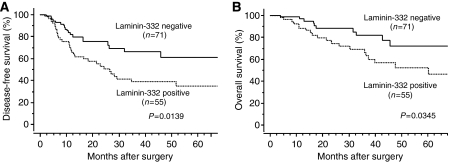
(**A**) Disease-free survival in relation to laminin-332 expression status of ESCC patients. (**B**) Overall cancer-specific survival in relation to laminin-332 expression status.

Patients with laminin-332-positive tumours had a significantly shorter disease-free survival and overall cancer-specific survival than patients with laminin-332 negative tumours (*P*=0.0139 and *P*=0.0345, respectively; Figure 2). In univariate analysis, the significant prognostic factors for predicting both the disease-free survival and overall cancer-specific survival were histological grading, T classification, TNM stage, lymph node metastasis and laminin-332 expression. In multivariate analysis, laminin-332 expression was the independent prognostic factors for predicting the disease-free survival, but not for the overall cancer-specific survival (Table 2).

### Expression of laminin-332 in oesophageal cancer cell lines

**Figure 3:**
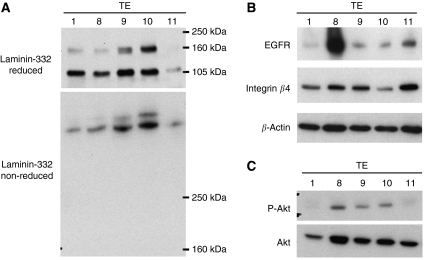
(**A**) Expression of laminin-332 in the conditioned medium of each ESCCs. All five ESCCs (TE1, 8, 9, 10, 11) secreted laminin-332, but the secreted level of TE11 is low. In the reduced medium (upper panel), the 155-kD band shows the un-cleaved laminin-332 *γ*2 chain, and the 105-kD band shows the cleaved laminin-332 *γ*2 chain. In the non-reduced medium (lower panel), the upper band shows the laminin-332 heterotrimer including the un-cleaved laminin-332 *γ*2 chain, and the lower band shows the laminin-332 heterotrimer including the cleaved laminin-332 *γ*2 chain. (**B**) Expression of EGFR and Integrin *β*4 in each ESCCs. All five ESCCs express EGFR. The band intensity shows the expression at the highest level in TE8 and at the lowest level in TE1. All five ESCCs express integrin *β*4 approximately at the same levels. (**C**) The activity of PI3K pathway was evaluated by Western blot analysis using the p-Akt antibody. The band intensity of p-Akt shows expression at higher level in TE8, TE9 and TE10 than in TE1, TE11.

A western blot analysis showed that all five ESCC cell lines (TE1, TE8, TE9, TE10, TE11) secreted laminin-332 *γ*2 chain into the conditioned medium, but the secreted level of TE11 was lowest (Figure 3A). In the reduced sample (upper panel), the 155-kDa band revealed the uncleaved laminin-332 *γ*2 chain, whereas the 105-kDa band revealed the cleaved laminin-332 *γ*2 chain. In the nonreduced sample (lower panel), the upper band revealed the laminin-332 heterotrimer including the uncleaved laminin-332 *γ*2 chain, whereas the lower band revealed the laminin-332 heterotrimer including the cleaved laminin-332 *γ*2 chain.

### Expression of EGFR and integrin *β*4 in oesophageal cancer cell lines

All five ESCCs expressed EGFR at the protein level. But the band intensity was different between each ESCCs, and showed expression at the highest level in TE8 and at the lowest level in TE1 (Figure 3B). All five ESCCs expressed integrin *β*4 at the protein level at the approximately same level (Figure 3B).

### PI3K activation in oesophageal cancer cell lines

We examined the activity of PI3K pathway by using the phosphorylated Akt (p-Akt) antibody by western blot analysis. The band intensity of p-Akt showed expression at higher level in TE8, TE9 and TE10 than in TE1, TE11 (Figure 3C).

### Laminin-332-blocking antibody inhibit PI3K activity and invasion in TE10

**Figure 4:**
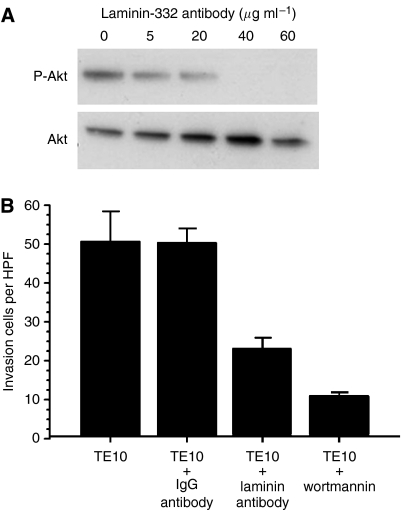
(**A**) The effect of the laminin-332 blocking antibody at different concentration on PI3K pathway in TE10 cell line. The laminin-332 blocking antibody suppresses the activation of PI3K pathway at the concentration of >40 *μ*g/ml. (**B**) The effect of the laminin-332 blocking antibody on the invasiveness of TE10. The number of invaded cells decreases to an approximately half with the laminin-332 blocking antibody (40 *μ*g/ml) by comparison with non-treatment or control IgG antibody (40 *μ*g/ml). The PI3K inhibitor (Wortmannin;100 nM) decreased to an approximately quarter by comparison with non-treatment.

The effect of the laminin-332-blocking antibody on PI3K pathway was tested *in vitro* using TE10, which secreted laminin-332 at the highest level among cell lines and had high PI3K activity *in vitro*. After the incubation with the laminin-332-blocking antibodies at different concentration for 24 h, the cells were solubilised. The laminin-332-blocking antibody suppressed the activation of PI3K pathway at the concentration of >40 *μ*g ml^−1^ (Figure 4A). The similar results were achieved by using TE8 and TE9 (data not shown). Next, we investigated whether the laminin-332-blocking antibody have an effect on the invasiveness of TE10 *in vitro* using the Matrigel-coated invasion chamber. The number of invaded cells decreased to an approximately half with the laminin-332-blocking antibody (40 *μ*g ml^−1^) by comparison with non-treatment or the IgG antibody (40 *μ*g ml^−1^). The PI3K inhibitor (Wortmannin;100 nM) decreased the number of invaded cells to an approximately quarter by comparison with non-treatment (Figure 4B).

### Laminin-332 promotes invasion via PI3K activation in TE11

**Figure 5:**
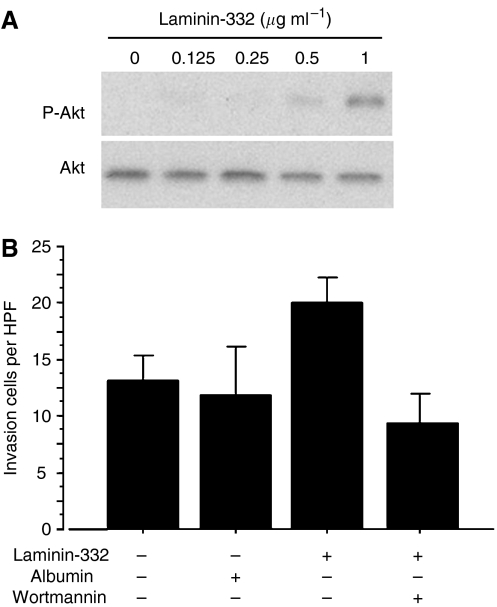
(**A**) The effect of the purified laminin-332 at different concentration on PI3K pathway in TE11 cell line. The addition of the purified laminin-332 activated the PI3K pathway at the concentration of >0.5 *μ*g/ml. (**B**) The effect of the purified laminin-332 on the invasiveness of TE11 cell line. The number of invaded cells increases with additional laminin-332 (0.5 *μ*g/ml) by comparison with non-treatment or control Albumin (0.5 *μ*g/ml). However, the number of invaded cells decreased with both additional laminin-332 (0.5 *μ*g/ml) and Wortomannin (100 nM).

The effect of the purified laminin-332 on PI3K pathway was examined *in vitro* using TE11, which secreted laminin-332 at the lowest level. After the incubation with the purified laminin-332 at different concentration for 6 h, the cells were solubilised. The laminin-332 activated the PI3K pathway at the proportion to the concentration of laminin-332 (Figure 5A). The number of invaded cells increased significantly with the purified laminin-332 (0.5 *μ*g ml^−1^) by comparison with non-treatment or control-purified albumin (0.5 *μ*g ml^−1^); however the addition of the PI3K inhibitor (Wortmannin;100 nM) decreased the number of invaded cells significantly (Figure 5B).

### Effect of PI3K inhibitor on the secretion of laminin-332 *in vitro*

**Figure 6:**
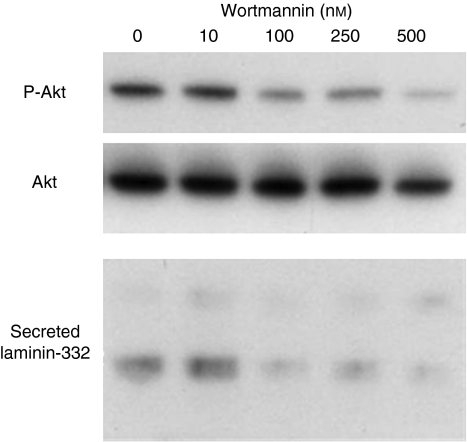
The relationship between the secretion of laminin-332 and PI3K pathway in TE10 cell line. The PI3K inhibitor (Wortmannin) decreases the activity of PI3K pathway in a dose-dependent manner, and the amount of the secreted laminin-332 into the medium decrease in proportion to the activity of PI3K pathway.

The relationship between the secretion of laminin-332 and PI3K pathway was also investigated. The PI3K inhibitor (Wortmannin) decremented the activity of PI3K pathway in a dose-dependent manner, and the amount of the secreted laminin-332 into the medium decreased in proportion to the activity of PI3K pathway (Figure 6).

## DISCUSSION

In the present study, the laminin-332 expression in cancer cells at the invasive front was seen in 44% of ESCC, and ESCC patients with its positivity had poorer prognosis than patients with its negativity. This result was consistent with previous reports about ESCC (Yamamoto *et al*, 2001; Fukai *et al*, 2005) and suggested that laminin-332 at the invasive front of ESCC might have an important role in the process of tumour invasion.

Laminin-332 has been reported to have potent cell migration-promoting activity. Miyazaki and co-workers showed that both soluble and deposited laminin-332 could stimulate the migration of human normal epithelial cells and cancer cells *in vitro* (Miyazaki *et al*, 1993; Kariya and Miyazaki, 2004). Laminin-332 has been reported to interact with integrin *α*3*β*1 or *α*6*β*4 and activate many signal mediators such as PI3K, focal adhesion kinase, protein kinase C, Rac, ERK, JNK and nuclear factor *κ*B, leading to enhanced cell migration and invasion (Nguyen *et al*, 2000; Kariya and Miyazaki, 2004; Nikolopoulos *et al*, 2005). In particular, the importance of PI3K activation induced by laminin-332 during carcinogenesis or tumour invasion has been recently suggested (Marinkovich, 2007; Waterman *et al*, 2007). Waterman and co-workers demonstrated that by *in vivo* model of human skin keratinocytes, the binding of laminin-332 to *α*6*β*4 integrin associated and drove tumorgenesis and invasion thorough PI3K activation in skin squamous cell carcinoma (Waterman *et al*, 2007). Furthermore, the interaction between laminin-332 *γ*2 chain III domain and EGFR has been suggested to have a role in PI3K activation via the action of FYN kinase (Schenk *et al*, 2003; Guo *et al*, 2006; Marinkovich, 2007). Briefly, the interaction of laminin-332 with *α*6*β*4 integrin and EGFR is involved in promoting PI3K activation and tumour invasion. To our knowledge, as there has been no article showing the relationship between laminin-332 and PI3K activation in ESCC, we focused on the relationship between the PI3K activation and invasiveness of ESCCs in the current study.

PI3K is a major signalling component downstream of growth factor receptor tyrosine kinase, and the PI3K pathway regulates various cellular processes, such as proliferation, growth, apoptosis and cytoskeleton rearrangement (Cantley, 2002). The aberrant activation of the PI3K pathway has been widely implicated in several types of carcinomas (Vivanco and Sawyers, 2002; Fresno Vara *et al*, 2004). In our *in vitro* study, the PI3K activity was assessed at higher level in TE8, TE9 and TE10 than in TE1 and TE11. As TE8, TE9 and TE10 secreted laminin-332 at higher level and also expressed EGFR and integrin *β*4, which were interacted with laminin-332, and TE11 secreted laminin-332 at very low level, the levels of PI3K activity in these ESCCs were understandable. Similarly in TE1, as this cell line expressed EGFR at very low level, the secreted laminin-332 might be unable to induce intracellular signal transduction *in vitro*. In fact, the addition of the purified laminin-332 did not activate the PI3K pathway in TE1 *in vitro* (data not shown). The laminin-332-blocking antibody deactivated the PI3K pathway, leading to suppress the invasiveness of TE10, which secreted laminin-332 at high level and had high PI3K activity. On the contrary, the addition of the purified laminin-332 activated the PI3K pathway, leading to increase the invasiveness of TE11, which secreted laminin-332 at lower level and had weak PI3K activity; however, the accession of the PI3K inhibitor decrease the invasiveness. These results suggest that laminin-332 could enhance the invasiveness of ESCCs through the PI3K activation.

In our *in vitro* study, the deactivation of PI3K pathway using PI3K inhibitor decreased the secretion of laminin-332, hence the PI3K activation was considered to be required for the secretion of laminin-332. Laminin-332 might provide the autocrine positive-feedback loop via the PI3K activation, contributing the invasive ability. These results suggest that the inhibitor of PI3K pathway could be useful as the anticancer therapy for ESCC (Luo *et al*, 2003). As to the downstream of PI3K pathway, further studies are needed.

In conclusion, the increased laminin-332 immunoreactivity is one of the prognostic factors of ESCC. Laminin-332 could provide the autocrine positive-feedback loop through the PI3K activation, contributing the invasive ability, hence the inhibitor of PI3K pathway might be useful as the anticancer therapies for ESCC.

## Figures and Tables

**Table 1:** The relationship between clinicopathological characteristics and expression of laminin 332

**Characteristic**	**Total (*n*=126)**	**Negative (*n*=71)**	**Positive (*n*=55)**	***P*-value**
Mean age		63.7±5.3	62.8±4.9	
				
*Sex*				0.81
Male	106	59	47	
Female	20	12	8	
				
*Tumour location*				0.62
Upper	27	13	14	
Middle	57	33	24	
Lower	42	25	17	
				
*Histological grading*				0.75
Well	51	29	22	
Moderate	48	26	22	
Poorly	27	16	11	
				
*T classification*				0.15
T1	63	39	24	
T2	24	15	9	
T3	39	17	22	
				
*N status*				0.14
N0	62	39	23	
N1	64	32	32	
				
*Stage*				0.036
I	47	30	16	
IIA	15	9	6	
IIB	31	20	11	
III	33	12	22	

N status indicates lymph node metastasis status.

*P*-values were calculated by using *χ*^2^ and Fisher's exact tests.

**Table 2:** Multivariate analysis of disease-free survival and overall cancer-specific survival in oesophageal squamous cell carcinoma

		**Disease-free survival**			**Overall survival**		
**Characteristic**	**No.**	**HR**	**95%CI**	***P***	**HR**	**95%CI**	***P***
*N status*							
N0 (reference)	62	1.00			1.00		
N1<	64	4.75	1.67–13.5	0.004	7.75	1.79–24.9	0.006
							
*Histological grading*							
Well (reference)	51	1.00			1.00		
Moderate, poorly	75	2.67	1.39–5.14	0.012	2.34	1.03–5.30	0.038
							
*Laminin 332*							
Negative (reference)	71	1.00			1.00		
Positive	55	2.01	1.18–2.64	0.043	1.93	0.97–3.42	0.062

HR=hazard ratio; CI=confidence interval.
